# ASO Author Reflections: The Safety and Efficacy of Robot-Assisted and Laparoscopic Distal Pancreatectomy in Patients with Resectable Left-Sided Pancreatic Cancer

**DOI:** 10.1245/s10434-023-13171-6

**Published:** 2023-02-06

**Authors:** Tess M. E. van Ramshorst, Jeffrey W. Chen, Mohammad Abu Hilal, Marc G. Besselink

**Affiliations:** 1 Department of Surgery, Amsterdam UMC, Location University of Amsterdam, Amsterdam, The Netherlands; 2 Cancer Center Amsterdam, Amsterdam, The Netherlands; 3 Department of General Surgery, Department of Hepato-biliary and Pancreatic Surgery, Istituto Ospedaliero Fondazione Poliambulanza, Brescia, Italy

## Past

Minimally invasive distal pancreatectomy (MIDP), consisting of both laparoscopic distal pancreatectomy (LDP) and robot-assisted distal pancreatectomy (RDP), is considered the preferable approach for benign lesions of the pancreatic body and tail, as also supported by two randomized trials.^1,2^ Lately, the indications of MIDP are broadening to malignant lesions^3^ and the use of the robot-assisted approach is emerging. Although several retrospective studies have compared LDP and open distal pancreatectomy (ODP) in patients with pancreatic cancer,^4^ studies assessing the safety and efficacy of RDP and LDP in patients with left-sided pancreatic cancer are lacking.

## Present

This large international multicenter study from 33 experienced centers analyzed the oncological and surgical outcome of 103 patients after RDP versus 439 patients after LDP for resectable left-sided pancreatic cancer. The R0-resection rate was comparable (75.7% RDP vs. 69.3% LDP, *p* = 0.404) whereas RDP was associated with an increased lymph node yield (18 vs. 16 nodes, *p* = 0.021). No differences were observed in overall survival and disease-free interval between RDP and LDP. Regarding surgical outcomes, RDP was associated with a longer operative time (290 vs. 240 min, *p* < 0.001), a lower conversion rate (4.9% vs. 17.3%, *p* = 0.001), more major complications (26.2% vs. 16.3%, *p =* 0.019), and longer hospital stay (10 vs. 8 days, *p* = 0.001). This largest retrospective cohort study to date comparing RDP with LDP in patients with resectable left-sided pancreatic cancer reflects the current practice in experienced centers, suggesting that RDP is as oncologically safe as LDP.^5^

## Future

To accurately assess the true impact of RDP and LDP on oncological and surgical outcome in patients with resectable left-sided pancreatic cancer, randomized trials are needed, which should take into account the current limitations of the retrospective design of this study and earlier studies. For example, resections should routinely include Gerota’s fascia, and the use of (neo)adjuvant treatment should be standardized. As prospective studies comparing RDP with LDP are lacking, future large, pragmatic randomized studies, possibly in a non-inferiority setting, should provide a definitive insight into the relative benefits and drawbacks of each approach. Finally, the results of the DIPLOMA-1 trial are expected soon, which compared MIDP (i.e., including both RDP and LDP) and ODP in patients with resectable left-sided pancreatic cancer and may confirm the oncological outcome of MIDP for this indication.
